# Specific Peptide from the Novel W-Tau Isoform Inhibits Tau and Amyloid β Peptide Aggregation *In Vitro*

**DOI:** 10.1021/acschemneuro.2c00188

**Published:** 2022-06-13

**Authors:** Raquel Cuadros, Mar Pérez, Daniel Ruiz-Gabarre, Félix Hernández, Vega García-Escudero, Jesús Avila

**Affiliations:** † Centro de Biología Molecular “Severo Ochoa” (CBMSO) CSIC/UAM, C/Nicolás Cabrera, 1, 28049 Madrid, Spain; ‡ Networking Research Centre on Neurodegenerative Diseases (CIBERNED), 28031 Madrid, Spain; § Departamento de Anatomía Histología y Neurociencia, Facultad de Medicina UAM, 28029 Madrid, Spain; ∥ Graduate Program in Neuroscience, Universidad Autónoma de Madrid (UAM), 28029 Madrid, Spain

**Keywords:** new tau isoform, w-Tau peptide, w-Tau peptide fragments, tau isoforms, amyloid peptide, aggregation

## Abstract

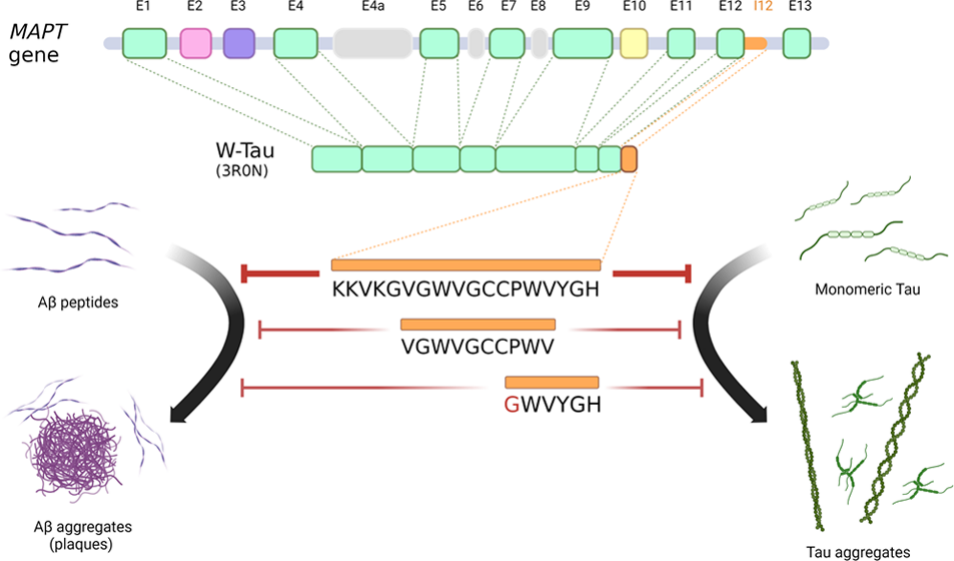

W-Tau, a new tau human-specific splicing isoform generated by intron retention, has been recently described. This isoform contains an 18-residue unique sequence corresponding to the translation of the retained region of intron 12. In this work, we have described that such 18-amino-acid peptide from the retained intron 12 can inhibit tau and β amyloid peptides aggregation under *in vitro* conditions. This inhibitory function is also present in smaller fragments of the 18-residue peptide.

## Introduction

Recently, a new tau isoform generated by the retention of intron 12 of the human *MAPT* gene has been described.^1^ Soon after the start of intron 12 of human *MAPT*, a stop codon appears, followed by a canonical polyadenylation sequence, resulting in the truncation of the protein at this point. Thus, this isoform differs from other human tau isoforms by lacking exon 13 of the *MAPT* gene and including an 18-amino-acid sequence corresponding to the translation of the retained fragment of the intron 12 in its place, at its carboxyl-terminal region, right after exon 12.^1^

The 18-residue sequence contains two tryptophan residues (W), an amino acid that cannot be found at any other point of the human tau sequence, and thus, the isoform has been named w-Tau. The 18-amino-acid peptide, termed in turn w-Tau peptide, may be involved in the decreased aggregation shown by w-Tau compared to that of other full-length and truncated tau isoforms.^1^

Indeed, analyzing the primary structure of the w-Tau peptide, some similarities were found with the sequences of a peptide family that prevents both tau and amyloid aggregation.^1,2^

In this short report, we have studied, *in vitro*, the inhibitory effect of the w-Tau peptide on the aggregation of full-length tau and on a self-aggregating tau fragment (residues 317–335 from full-length 4R2N Tau).^3,4^ In parallel, the inhibition of the aggregation of β amyloid peptide by w-Tau peptide was also analyzed.

## Materials

Heparin sodium salt from porcine intestinal mucosa was obtained from Sigma (H3393-100KU).

### Isolation of Tau Protein

Recombinant human Tau 3R or 4R containing three or four tubulin-binding motifs, respectively, was expressed and purified as previously reported.^5,6^

### Synthesis of Tau Peptides and β Amyloid Peptide

The tau peptide 317–335aa (1/2R peptide) (^317^KVTSKCGSLGNIHHKPGGG^335^) was purchased from NEOSYSTEM LABORATOIRE (STRASBOURG, FRANCE). The tau peptide 387–393 (^387^DHGAEIV^393^), the w-Tau peptide, and its fragments: full 18aa (KKVKGVGWVGCCPWVYGH), 10aa (VGWVGCCPWV), 6aa (GWVYGH), and the β-amyloid peptide (residues 25–35) (GSNKGAIIGLM) were obtained from ABYNTEK BIOPHARMA S.L. (Parque Tecnológico de Bizkaia. DERIO, Spain). All of the peptides were dissolved in sterile Milli-Q distilled water.

## Methods

### *In Vitro* Polymerization Analyses: Tau Protein, β Amyloid Peptide

Tau protein and its peptides were dissolved at a concentration of 10 mg/mL in distilled water, aliquoted, and immediately used or frozen to be used only once, to avoid several freezing/thawing cycles. Amyloid β peptide was stored in small aliquots as a solid at 4 °C.

Recombinant human Tau 3R or 1/2R peptide (10 μg) was incubated in 10 μL of Buffer A (0.1 MES (pH 6.4), 0.5 mM MgCl_2_, and 2 mM EGTA) and 0.5 M NaCl, in the absence or presence of different concentrations of heparin. The optimal Tau protein:heparin ratio to visualize fibrillar polymers was 1:4 (mass/mass). For the 1/2R tau peptide, the optimal peptide:heparin ratio was found to be 1:1 (mass/mass). To study the effect of the 18aa Tau peptide on the polymerization of Tau 3R, equimolar amounts of Tau and peptide were used. To analyze the effect of w-Tau (18aa and fragments) peptides on 1/2R tau peptide polymerization, w-Tau peptides were added at final concentrations of 1, 0.1, and 0.01 μg/μL. The reactions were allowed to proceed at room temperature for 7 days before analysis. In the presence of heparin, protein assembly is accelerated and reaction conditions could be set at 3 days at a lower temperature (4 °C).^4^ Tau polymers were partially quantified by observing filamentous polymers at electron micrographs of several fields. For a proper quantification of Tau proteins, the protein aggregates were pelleted by centrifugation at 28 psi for 30 min, using a table Beckman Airfuge ultracentrifuge, and the pelleted protein was analyzed by Western blot using tau antibody 7.51.^7^

As for the corresponding amyloid β peptide polymerization assays, 10 μL of w-Tau peptide at a concentration of 3, 0.3, or 0.03 μg/μL in buffer A was added to 10 μg of lyophilized amyloid β peptide. After 10 min of incubation at room temperature, fibrillar polymers in the absence or presence of w-Tau peptides can be visualized by electron microscopy.

Amyloid β polymers quantifications were done by pelleting the polymers, as indicated for tau aggregates, and measuring protein amount by dot blot using Coomassie blue.^8^

### Electron Microscopy Analysis

Polymerization reaction samples were added to a Formvar (400 mesh) carbon-coated grid for 5 min. The grid was stained with 2% uranyl acetate for 40 s. The grids were examined with a JEM1010 (Jeol) transmission electron microscope. Images were taken with a TemCam F416 (TVIPS) camera at a magnification of 20,000×.

## Results

### w-Tau Peptide Inhibits the Polymerization of Tau Protein *In Vitro*

**Figure 1:**
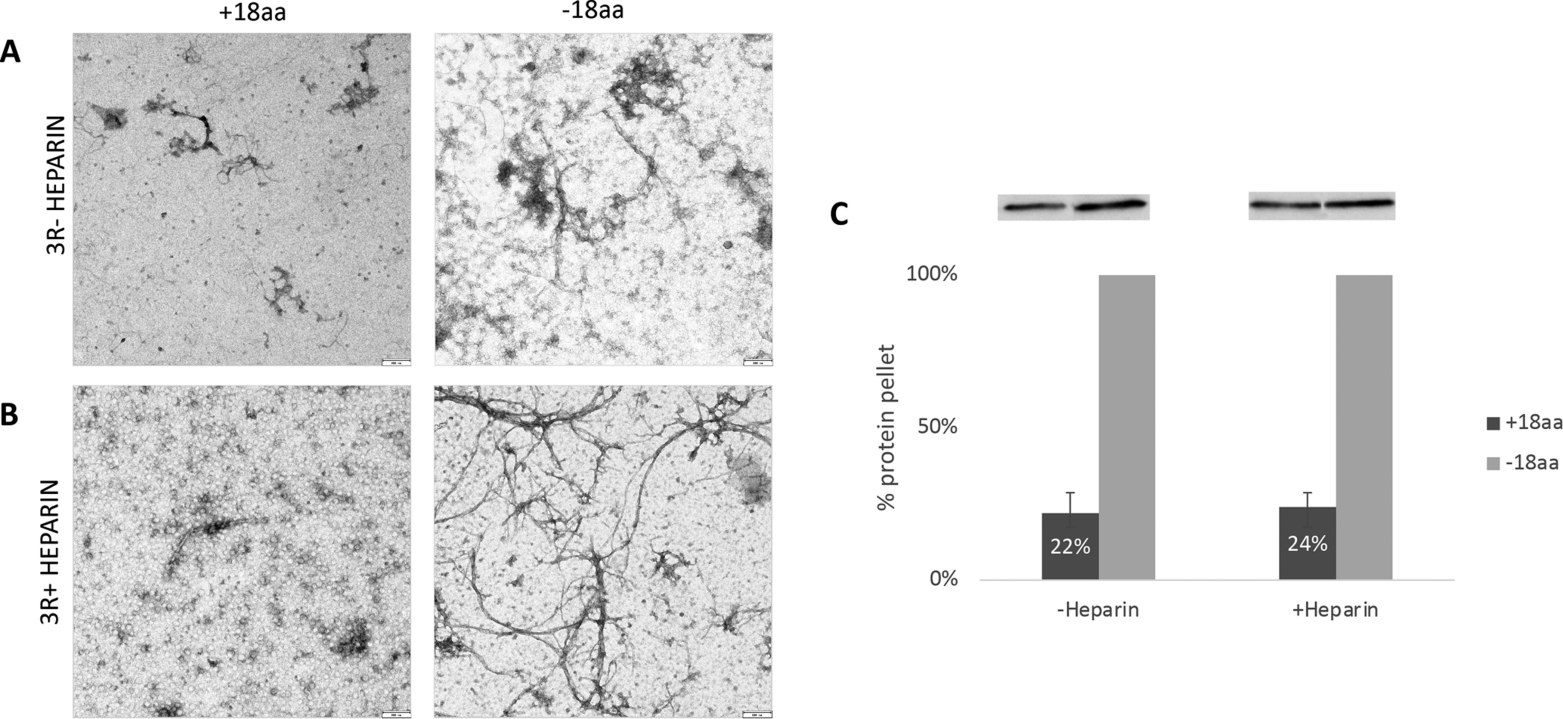
w-Tau peptide prevents 0N3R Tau polymerization in the absence or presence of heparin. (A) Tau (0N3R) polymerization in the absence of heparin and the absence of w-Tau peptide (right) and in the absence of heparin and the presence of w-Tau peptide (left); on a 1:1 tau/w-tau peptide molar ratio. (B) Tau polymerization in the presence of heparin and the absence of w-Tau peptide (right) or the presence of w-tau peptide (left); on a 1:1 tau/w-tau peptide molar ratio. (C) Quantification of the results found in (A) and (B), after centrifugation of the polymerized protein, electrophoresis of the pelleted protein, and recognition of Tau protein by WB, using tau antibody 7.51 (see Methods).

It was already described that highly purified tau protein can polymerize *in vitro*,^9^ yielding filaments similar to those found in the brain of Alzheimer’s disease patients,^10^ but not identical.^11,12^ Also, it is known that, in the presence of heparin, filament polymerization accelerates for ON3R tau isoform.^3,4,13^ Thus, we have tested the action of w-Tau peptide on tau protein aggregation in the presence or absence of heparin. Figure 1 shows that, in both situations, w-Tau peptide (18aa) inhibits tau filament assembly. When 0N4R tau isoform was tested, inhibition of its polymerization by w-Tau peptide was found as well (Supporting Information 1).

### Assembly of Tau Peptide (317–335aa) Is Inhibited by w-Tau Peptide

**Figure 2:**
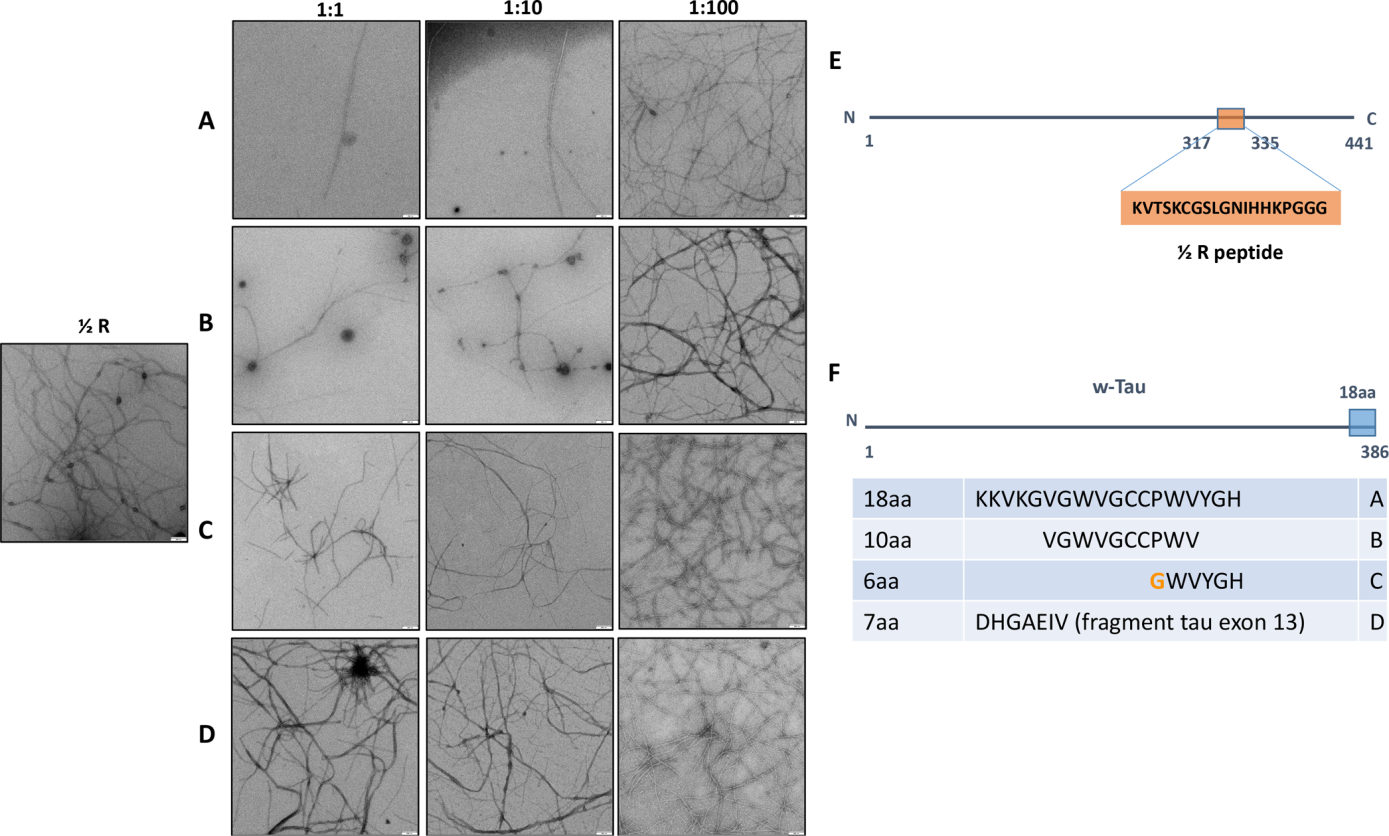
w-Tau peptide and its fragments prevent, in a dose-dependent manner, the polymerization of 1/2R Tau peptide. (A) Effect of increasing amounts of w-Tau peptide (18aa) on 1/2R Tau polymerization. (B) Effect of increasing amounts of w-Tau peptide fragment (10aa) on 1/2R Tau polymerization. (C) Effect of increasing amounts of w-Tau peptide fragment (6aa) on 1/2R Tau polymerization. (D) Effect of increasing amounts of tau peptide at the C-terminal region (7aa) on 1/2R Tau polymerization. This peptide (7aa) was used as a negative control. (E) Schematic representation of 2N4R tau molecule showing the localization of 1/2R peptide. (F) Schematic representation of w-Tau molecule containing its 18aa peptide at the C-terminal. The sequence of this 18aa peptide and some of its fragments are indicated. In the case of the 6aa fragment, P was changed by G. The 7aa peptide corresponds to residue 287–293 of 2N4R tau molecule.

In a pioneer study, it was shown that the microtubule-binding region of tau protein, containing similar, but not identical, three or four repeated sequences was the region involved in tau–tau interaction.^14^ Focusing on the third repeat (residues 306–335), it was found that it may be able to self-assemble into filamentous peptides.^3^ Even small fragments of that 306–335 peptide, like peptides comprising 306–311^15^ or that containing residues 317–335 (1/2R peptide)^3,4^ are able to self-polymerize. Figure 2 shows the dose-dependent inhibition of the tau 1/2R peptide (317–335) polymerization mediated by the presence of w-Tau peptides.

### Fragments of w-Tau Peptide Could Inhibit Tau Polymerization

Since w-Tau peptide contains 18aa (KKVKGVGWVGCCPWVYGH), we have tested if fragments of this peptide like a 10aa peptide (VGWVGCCPWV) or a 6aa peptide (**G**WVYGH) can also inhibit the self-assembly of the 1/2R tau fragment (317–355). Figure 2B,C indicates that also those peptides could inhibit in a dose-dependent manner this fragment’s assembly. As a negative control, Figure 2D indicates the lack of inhibition of tau assembly in the presence of the peptide DHGAEIV, a peptide containing the residues 387–393 of the 2N4R tau molecule.

### w-Tau Peptide Inhibits Amyloid β Peptide Assembly

**Figure 3:**
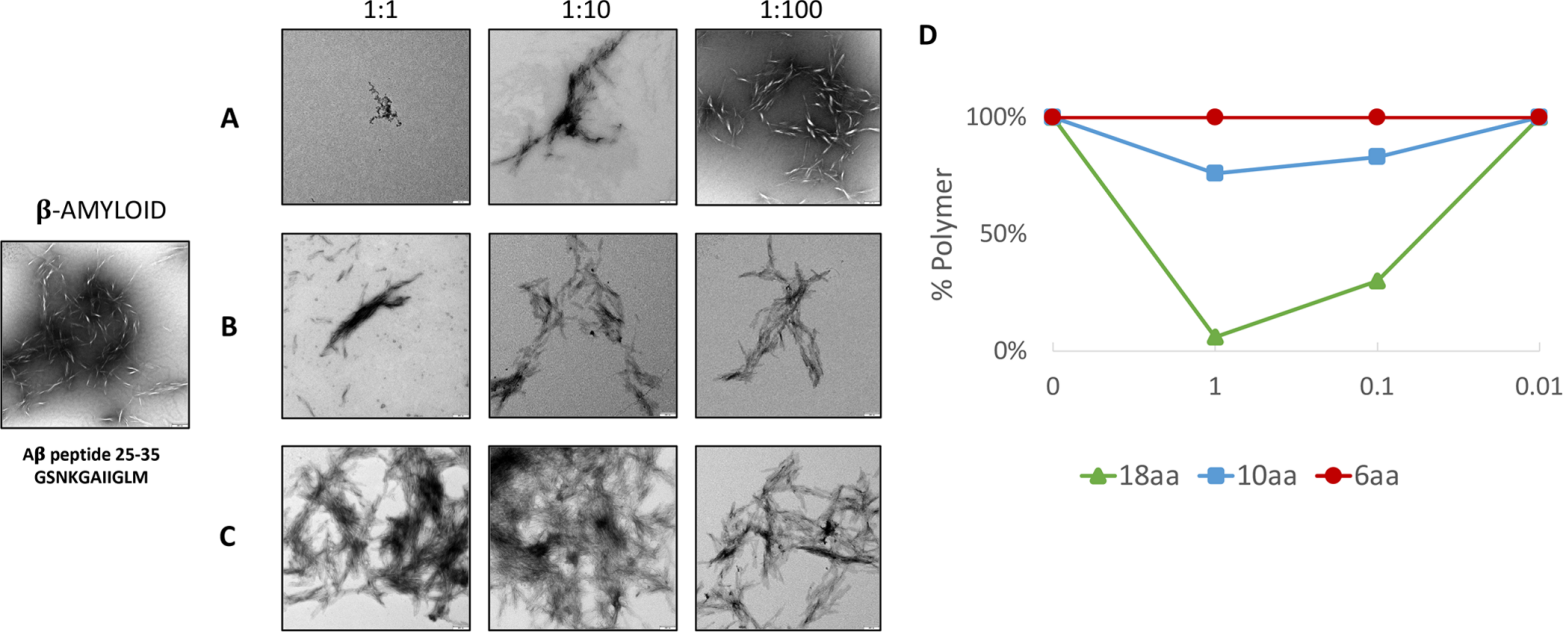
Effect of w-Tau peptide and its fragments on amyloid β polymerization. (A) Effect of w-Tau peptide (18aa) on amyloid β polymerization. (B) Effect of w-Tau peptide fragment (10aa) on amyloid β polymerization. (C) Effect of w-Tau peptide fragment (6aa) on amyloid β polymerization. (D) Quantification of the whole polymers was done as indicated in the Methods section, at different amyloid/w-Tau peptides ratios.

The sequence of w-Tau peptide is similar to that of the LYIWVQ family peptides that prevent the assembly of tau and amyloid peptide.^2^ Thus, we have tested if w-Tau peptides might also prevent amyloid peptide assembly (Figure 3). To do that, w-Tau peptides at different concentrations in buffer A, or buffer A alone as a control, were added to a known amount of lyophilized (solid) β amyloid peptide and properly mixed. The polymerized protein in an aliquot of the resulting preparation was visualized by electron microscopy. The rest of the preparation was subjected to centrifugation, and the pelleted (polymerized/aggregated) protein was measured by electrophoresis. Figure 3A shows that 18-aa w-Tau peptide inhibits amyloid polymerization in a dose-dependent manner. In addition, 10-aa (Figure 3B) fragments but not 6-aa fragments (Figure 3C) of 18-aa w-Tau peptide also slightly inhibit the formation of amyloid polymers.

## Discussion

In this work, we have shown that w-Tau peptide, the sequence of intron 12 retained in the new w-Tau isoforms,^1^ is able to inhibit tau protein assembly. This feature could explain the decreased capacity of w-Tau isoform for self-assembly^1^ and suggests its action as a potential inhibitor for the assembly of the other tau isoforms. The inhibitory role of w-Tau peptide not only for tau protein assembly but also for the inhibition of amyloid β peptide polymerization may suggest a potential use as a tool to prevent tauopathies that are mainly characterized by the presence of aberrant tau protein aggregates.^16,17^ The inhibition of amyloid aggregation could be also of interest for the most relevant tauopathy, Alzheimer’s disease.

## Supporting Information Available

Electron microscopy images of 0N4R Tau polymerization (PDF)
