# Supplementary material for: Specific Peptide from the Novel W-Tau Isoform Inhibits Tau and Amyloid β Peptide Aggregation In Vitro
Source: ACS Chem Neurosci. 2022 Jun 13;13(13):1974–8. doi: 10.1021/acschemneuro.2c00188 (PMC9264359; doi:10.1021/acschemneuro.2c00188)
Supplement: Supplementary file 1 — cn2c00188_si_001.pdf [file cn2c00188_si_001.pdf]

A specific peptide from the novel W-tau isoform inhibits tau and amyloid beta peptide aggregation *in vitro*

Raquel Cuadros[1,2], Mar Pérez[3], Daniel Ruiz-Gabarre[1,4], Félix Hernández[1], Vega García-Escudero[2,3,4] & Jesús Avila[1,2,*]

[1] Centro de Biología Molecular "Severo Ochoa" (CBMSO) CSIC/UAM, Madrid, Spain.
[2] Networking Research Centre on Neurodegenerative Diseases (CIBERNED), 28031 Madrid, Spain.
[3] Departamento de Anatomía Histología y Neurociencia, Facultad de Medicina UAM, Madrid, Spain
[4] Graduate Program in Neuroscience, Universidad Autónoma de Madrid (UAM), 28029 Madrid, Spain

[*] Correspondence: Jesús Avila, Centro de Biología Molecular Severo Ochoa (CSIC- UAM), C/ Nicolás Cabrera, 1. 28049 Madrid, Spain. E-mail: javila@cbm.csic.es. Telephone number: +34 911964564

Contents

1. Electron microscopy of 0N4R Tau polymerization

W-Tau peptide prevents 0N4R Tau polymerization

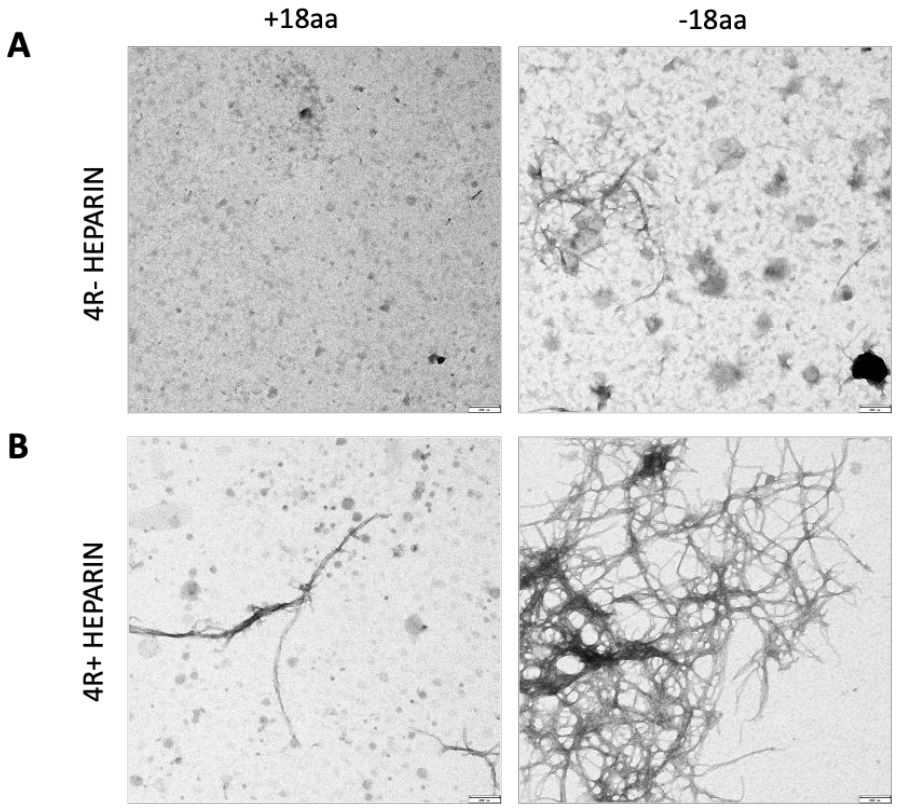

Figure S1: W-Tau peptide prevents 0N4R Tau polymerization. Analogous analysis to that shown in Figure 1 A & B, but testing 0N4R tau isoform instead of 0N3R tau isoform.
